# Supplementary material for: Survival after Lung Metastasectomy from Esophageal Cancer: Results from a Multi-Institutional Database
Source: Cancers (Basel). 2023 Feb 25;15(5):1472. doi: 10.3390/cancers15051472 (PMC10000551; doi:10.3390/cancers15051472)
Supplement: Supplementary file 1 [file cancers-15-01472-s001.zip › cancers-2138224-File S1.pdf]

Membership of Metastatic Lung Tumor Study Group of Japan

Affiliation List

- Department of Thoracic Surgical Oncology, Cancer Institute Hospital
- Division of Thoracic Surgery, Department of Surgery, Keio University School of Medicine
- Department of General Thoracic Surgery, Saitama Medical Centre, Saitama Medical University
- Division of Thoracic Surgery, Chiba Cancer Centre
- Department of General Thoracic Surgery, Chiba University Graduate School of Medicine
- Department of Thoracic Surgery, Tokyo Medical University
- Department of Thoracic Surgery, Graduate School of Medicine, The University of Tokyo
- Division of Thoracic Surgery, Tochigi Cancer Centre
- Section of Chest Surgery, Fukujuji Hospital, Japan Anti Tuberculosis Association
- Department of Thoracic Surgery, National Defense Medical College
- Department of Thoracic Surgery and Thyroid Surgery, Kyorin University School of Medicine
- Division of Chest Surgery, Department of Surgery, School of Medicine, Toho University
- Respiratory Medicine, National Hospital Organization Tokyo Medical Center
- Department of Thoracic Surgery, Yamagata Prefectural Central Hospital
- Department of Thoracic Surgery, Kimitsu Central Hospital
- Department of Respiratory Surgery, Nihonkai General Hospital
- Department of Surgery, Teikyo University School of Medicine
- Department of General Thoracic Surgery, Osaka University Graduate School of Medicine
- Department of Thoracic Surgery, Aichi Cancer Center Hospital
- Department of Thoracic Surgery, Hakodate Goryoukaku Hospital
